# A label-free aptasensor for the detection of ATP based on turn-on fluorescence DNA-templated silver nanoclusters†

**DOI:** 10.1039/d2ra04636a

**Published:** 2022-10-20

**Authors:** Baozhu Zhang, Ziyao Yang, Yuxia Li, Ling Ma, Fenfang Li, Xiuqing Lv, Guangming Wen

**Affiliations:** Department of Chemistry and Chemical Engineering, Jinzhong University Yuci 030619 P. R. China zhangbaozhu518@126.com wgm@sxu.edu.cn

## Abstract

A label-free aptasensor has been fabricated in order to detect adenosine triphosphate (ATP) using turn-on fluorescence DNA-Ag NCs. The fluorescence of the DNA-Ag NCs could increase remarkably with the addition of ATP mainly because ATP specifically interacts with its aptamer to change the microenvironment of the darkish DNA-Ag NCs located at one terminus or two termini due to the conformational alteration of the aptamer structure. The proposed sensor can detect ATP in a linear range of 6–27 mM with a good detection limit of 5.0 μM. Additionally, the proposed method succeeded in detecting ATP in fetal bovine serum.

## Abstract 2

A label-free aptasensor has been fabricated in order to detect adenosine triphosphate (ATP) using turn-on fluorescence DNA-Ag NCs.

## Graphical abstract

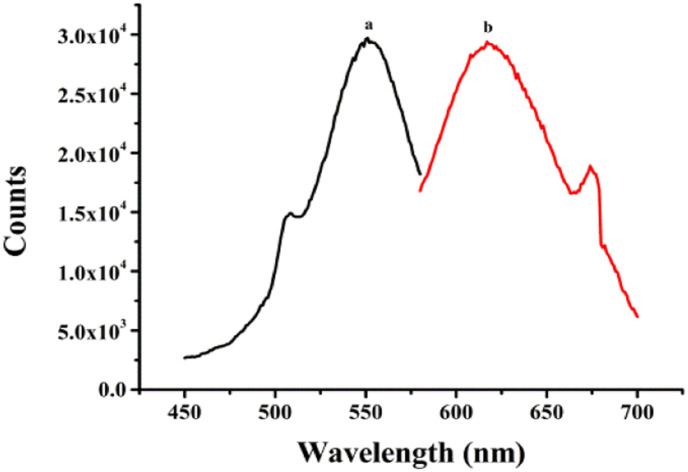

## 1 Introduction

Nanotechnology, which focuses on structures of matter on the order of less than 100 nm, has been paid more attention recently. In particular, nanoparticles are considered unique due to their small size, high surface area, and surface chemistry and charge, and therefore they are famous for their multi-functionality and are applied in many fields.^1^ Among them, metal (Cu, Ag, Au, *etc.*) nanoclusters (NCs), comprising several to hundreds of metal atoms and with diameters less than 2 nm, have attracted considerable research interest as various chemical or biological analytes can be detected using them as fluorescent systems.^2–4^ Particularly, DNA-templated metal NCs exhibit better inherent properties, such as high photostability, biocompatibility, low toxicity, large Stokes shift and adjustable light emission wavelength, than organic quantum dots or fluorophores.^5–7^ All kinds of applications based on DNA-templated metal NCs including optoelectronic devices,^8^ biological imaging,^9^ optical sensing,^10^ and in the clinical field have been developed in recent years. In addition, some metal and metal oxides conjugated to form nanocomposites with silver nanoparticles or with other materials have been applied in catalysis;^11^ the agricultural industry;^12^ the biomedical domain, including in diagnosis and therapeutics, biosensing, bioimaging devices, drug delivery systems, and bone substitute implants;^1,13^ and in the management and treatment of metabolic syndrome; *etc.*^14^

ATP is utilized as an energy currency in living organisms^15^ and plays a key role in the regulation of biochemical pathways and cellular metabolism in cell physiology.^16,17^ Furthermore, ATP has been utilized as an indicator for ischemia, Alzheimer's disease, hypoglycemia, Parkinson's disease, and so on.^18^ Therefore, various detection methods of ATP have been developed, which include the colorimetric method,^19^ fluorescence analysis,^20^ electrochemical analysis,^21^ and liquid chromatography.^22,23^ However, these detection methods are time-consuming and laborious due to their complex sample preparation procedures and/or modification of electrodes and fluorescent probes. In recent years, fluorescence detection methods have been applied to analyze ATP with simple operation as well as good sensitivity and selectivity.^20,24^ Since the selectivity and sensitivity mainly depend on the performance of the probe,^25^ it is very significant to construct superior ATP probes for the improvement of their selectivity and sensitivity.

**Scheme 1:**
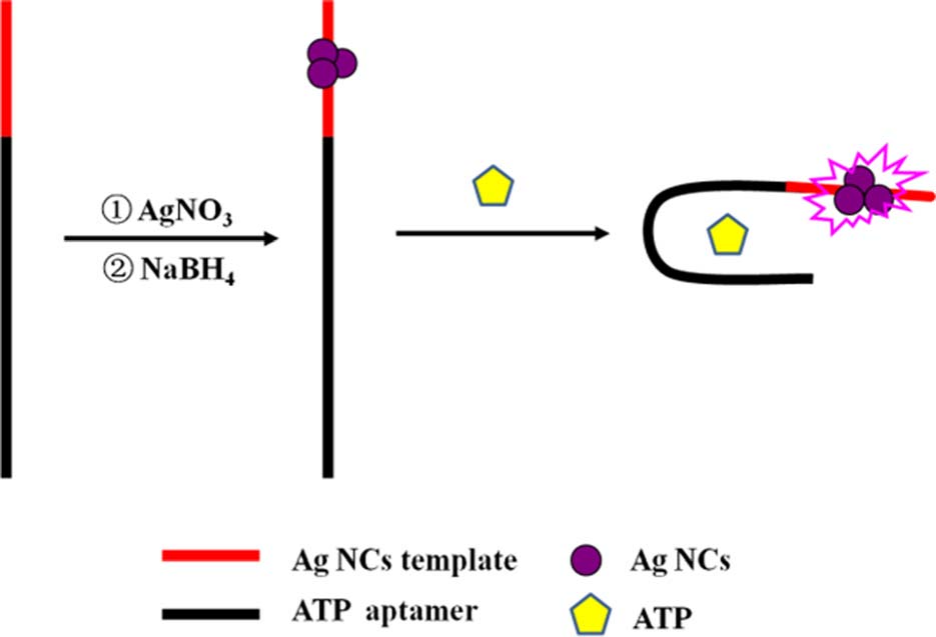


It has been reported that non-emissive DNA-Ag NCs can turn into bright emitters after being placed in the environment of a guanine-rich (G-rich) DNA sequence, and their fluorescence can also increase evidently when two darkish DNA-Ag NCs are close to each other using their complementary linkers.^26^ Inspired by this concept, an aptasensor strategy was developed based on ATP aptamer DNA-templated Ag NCs. The mechanism is illustrated in Scheme 1. The DNA template is composed of an ATP aptamer and Ag NC-nucleation sequences located at the left and right, respectively. The darkish DNA-Ag NCs at the right of the templates become bright emitters under the alteration of the microenvironment due to the conformational change of ATP aptamer as ATP is added. Therefore, the designed DNA-Ag NCs aptasensor affords the turn-on, sensitive, and convenient analysis of ATP.

## 2 Experimental

### 2.1 Materials and apparatus

Oligonucleotides, ATP, uridine triphosphate (UTP), cytidine triphosphate (CTP), and guanosine triphosphate (GTP) were provided by Sangon Biotechnology Inc. (Shanghai, China), and the sequences and names of the DNA are listed in ESI Table S1.† Fetal bovine serum was obtained from Yuanye Biotechnology Co. Ltd. (Shanghai, China). Silver nitrate (AgNO_3_, 99.8%) and sodium borohydride (NaBH_4_, 98%) were purchased from Aladdin Biochem Technology Co. Ltd. (Shanghai, China). All the chemical reagents were of analytical grade and used as received without further purification. Phosphate-buffered saline (PBS, 20 mM, pH 7.0) was used in all of the experiments. All solutions were prepared using Milli-Q water (18.2 MΩ cm).

Fluorescence measurements were performed on a Edinburgh FS5 fluorescence spectrophotometer (Edinburgh Instruments, Livingston, UK) at ambient temperature, and the excitation and emission slit widths were 2.0 nm and 4.0 nm, respectively. UV-vis absorption spectra were recorded on a Cary 50 Bio spectrophotometer (Varian Inc., CA) at room temperature. The average size and morphologies of the Ag NCs were determined using a JEOL JEM-2100 transmission electron microscope with an acceleration voltage of 200 kV. Time-resolved fluorescence measurements were performed using an FS5 fluorescent lifetime spectrometer operating in the time-correlated single photon counting (TCSPC) mode using a semiconductor laser (405 nm) as the excitation source. The commercial software by Edinburgh Instruments was used for data analysis. When 
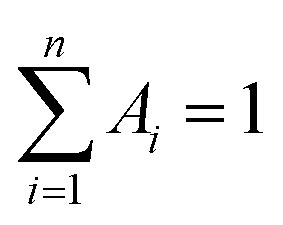
, the average excited state lifetime is expressed by the equation 
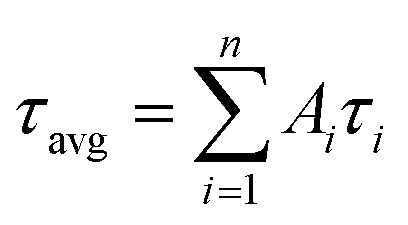
. The reported spectrum of each sample represents the average of three scans. X-Ray photoelectron spectroscopy (XPS) (ESCAL-ab 220i-XL, VG Scientific, England) was performed using monochromatic Al Kα as a source at 1486.6 eV.

### 2.2 Synthesis of Ag NCs

DNA-Ag NCs were synthesized according to the reported literature.^27^ A schematic illustration of the synthesis of the Ag NCs is presented in ESI Fig. S1.† Firstly, DNA (3.0 μM) and Ag NO_3_ (18 μM) were added to a PBS solution (20 mM, pH 7.0) in sequence with uniform mixing. After the mixed solution were kept from light and incubated at 4 °C for 20 min, fresh NaBH_4_ (18 μM) was added to the as-prepared solution and stirred vigorously for at least 1 min. The mixture was incubated and kept away from light at 4 °C for about 1 h to obtain DNA-Ag NCs for further use.

### 2.3 Assay of ATP

ATP of different concentrations (0–33 mM) was sequentially added to the as-synthesized DNA-Ag NCs solution. The fluorescence spectra of the above solution were recorded at room temperature. ATP and its analogues, including GTP, UTP, and CTP, were determined using the same method as ATP in order to measure the specificity of the proposed sensor for ATP.

### 2.4 Application of the aptasensor

The practicability of the proposed aptasensor was further investigated. ATP at different concentrations was spiked in 100-times-diluted fetal bovine serum solution and measured using the same method as the detection of ATP.

## 3 Results and discussion

### 3.1 Optical characterization of DNA-Ag NCs

**Fig. 1:**
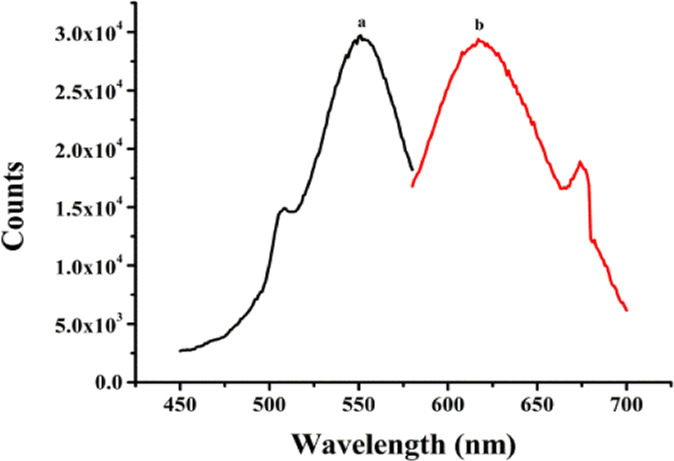
The excitation (a) and emission (b) spectra of (L)BT5A5-Ag NCs.

The secondary structures and base sequences of DNA templates play a key role in determining the optical properties of metal NCs.^28,29^ Thus, three DNA templates named BT5A5, (L)BT5A5, and BT5A5(R) were designed, and their sequences are listed in ESI Table S1.† Among them, the aptamer of ATP (italic) is in the middle, while the C-rich segment-protected DNA-Ag NCs are at the 5′ and/or 3′ ends (bold). (L)BT5A5 and BT5A5(R) are derived from BT5A5, which are deleted from the left (or right) C-rich segments and the TTTTT (or AAAAA) linker (underline) of BT5A5. The results of the optical characterization of (L)BT5A5 are shown in Fig. 1. The excitation and emission spectra are represented by curves (a) and (b) in Fig. 1, and the excitation and emission peaks are at 550 and 620 nm, respectively. Similarly, the excitation and emission spectra of BT5A5 and BT5A5(R)-Ag NCs are displayed in ESI Fig. S2A and B.† The excitation and emission wavelengths are 550 and 610 nm for BT5A5-Ag NCs, and 557 and 628 nm for BT5A5(R)-Ag NCs, respectively. The UV-Vis absorption spectra of (L)BT5A5-Ag NCs were measured in the absence and presence of ATP. There are two peaks in each of the absorption spectra of ESI Fig. S3;† one peak at 430 nm belongs to the characteristic plasmon absorption band of Ag nanoparticles and the other peak at 550 nm should be attributed to the absorption band of Ag NCs.^30^ Therefore, (L)BT5A5-Ag NCs contains Ag nanoparticles. The absorption intensity becomes stronger with an increasing concentration of ATP. This demonstrates that the amount of (L)BT5A5-Ag NCs gradually increases.

The relationship between the fluorescence intensity changes of the three DNA-Ag NCs and storage time was determined due to the great influence of the stability of the sensor on its detection performance. As displayed in ESI Fig. S4,† the fluorescence intensity of (L)BT5A5-Ag NCs reached a plateau after 4 h, and was then stable for about 2 h. However, the fluorescence intensities of BT5A5-Ag NCs and BT5A5(R)-Ag NCs decrease with storage time. Hence, the stability of (L)BT5A5-Ag NCs is superior to that of the others.

### 3.2 Characteristics of (L)BT5A5-Ag NCs

**Fig. 2:**
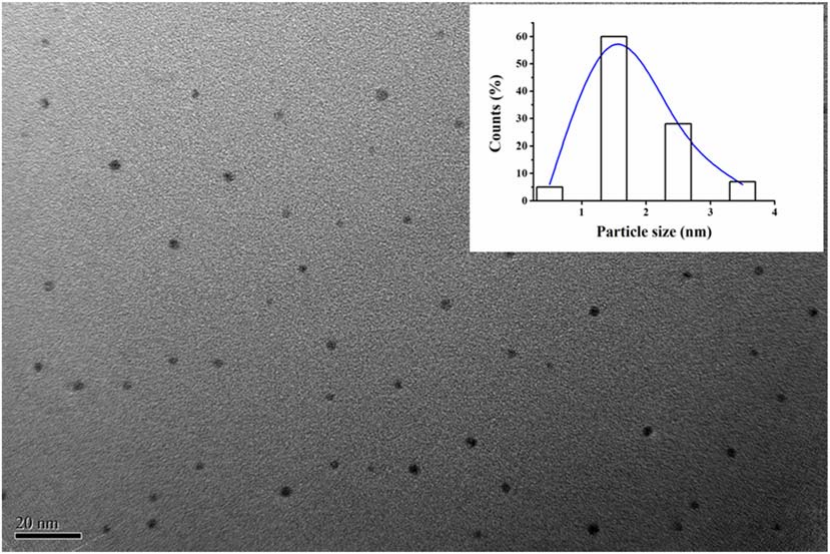
TEM image of (L)BT5A5-Ag NCs. The inset is the size distribution histogram fitted by Lorentzian function.

The size of (L)BT5A5-Ag NCs was measured using transmission electron microscopy. As illustrated in Fig. 2, uniform Ag NCs in the range of 1 to 3 nm are distributed, and the average particle size of the Ag NCs is about 2 nm, which is in accordance with the sub-2 nm size of metal NCs,^31^ which was obtained using a histogram fitted by the Lorentzian function.

**Fig. 3:**
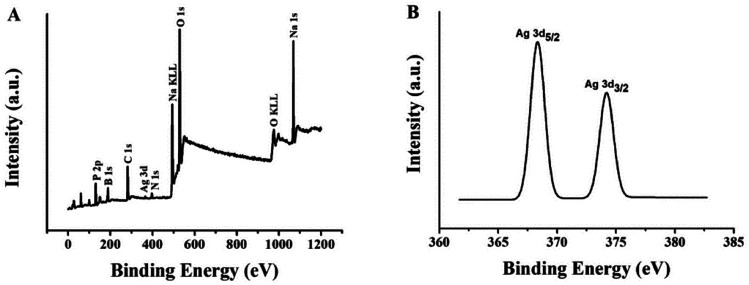
(A) XPS spectrum of (L)BT5T5-Ag NCs. (B) Ag 3d region of the XPS spectrum of (L)BT5T5-Ag NCs.

In order to analyze the valence state of the Ag element and the existence of various elements in (L)BT5A5-Ag NCs, the XPS wide scan survey spectra and Ag 3d spectra were recorded. Fig. 3A shows at least 10 peaks, of which 9 corroborate that P, B, C, Ag, N, Na and O are present. There are 2 peaks in the expanded spectrum of Ag 3d (Fig. 3B), the binding energy values of which are 368.3 eV for Ag 3d_5/2_ and 374.2 eV for Ag 3d_3/2_, which could further verify the existence of Ag(0) in (L)BT5A5-Ag NCs.^32,33^

### 3.3 Optimization of the experimental conditions for ATP determination

#### 3.3.1 Response of different DNA-Ag NCs to ATP

The properties of metal NCs are affected by the base sequences and secondary structures of DNA templates.^30,34^ The fluorescence change of the DNA-Ag NCs stabilized by three DNA oligonucleotides was investigated upon the addition of ATP. As displayed in ESI Fig. S5,† the fluorescence intensities of the three DNA-Ag NCs all increasing on adding ATP. The relative fluorescence intensity (*F*/*F*_0_, where *F*_0_ and *F* are the fluorescence intensities of the Ag NCs with no addition of and after adding 21 mM ATP, respectively) of (L)BT5A5-Ag NCs increases most obviously among these, with an *F*/*F*_0_ equal to ∼30. Therefore, (L)BT5A5-Ag NCs is the perfect candidate for the following experiments.

#### 3.3.2 Determination of the incubation time of (L)BT5A5-Ag NCs with ATP

In order to detect ATP more sensitively with the proposed sensor, the optimal reaction time for (L)BT5A5-Ag NCs with ATP was evaluated. As shown in ESI Fig. S6,† the fluorescence intensity of (L)BT5A5-Ag NCs reaches the plateau in 35 min and hardly changes after 35 min. Thus, 35 min is regarded as the optimal reaction time for the sensor with ATP.

#### 3.3.3 Optimization of pH

The value of pH has a great effect on the detection of ATP. The fluorescence intensity of (L)BT5A5-Ag NCs was tested in different pH values. As shown in ESI Fig. S7,† the relative fluorescence intensity (*F*/*F*_0_, where *F*_0_ and *F* are the fluorescence intensities of the Ag NCs with no addition of and after adding 12 mM ATP, respectively) of (L)BT5A5-Ag NCs increases from pH 5 to 7, plateaus at pH 7, and then declines from pH 7 to 9. Therefore, pH 7 was chosen as the optimal value for all the experiments.

### 3.4 Assay of ATP

**Fig. 4:**
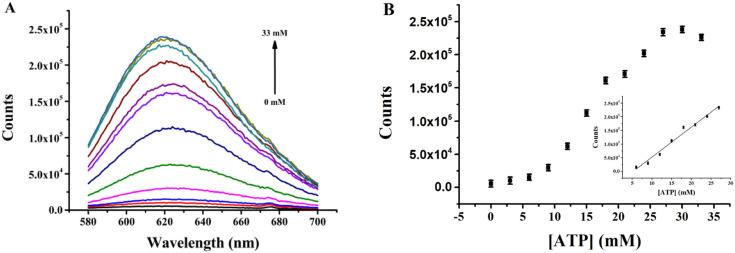
(A) Fluorescence spectra of (L)BT5A5-Ag NCs upon the addition of ATP (0–33 mM). (B) The relationship between the fluorescence intensity and the concentration of ATP. Inset: the relationship between the fluorescence intensity and the concentration of ATP. Error bars represent the standard deviation of three parallel experiments.

Quantitative analysis of ATP utilizing the (L)BT5A5-Ag NCs sensor was investigated under the optimized experimental conditions. The fluorescence intensities of (L)BT5A5-Ag NCs were determined with varying concentrations of ATP from 0 to 33 mM. As shown in Fig. 4A and B, a gradual enhancement in fluorescence intensity is observed with increasing concentration of ATP, with a good linear relationship (*R*^2^ = 0.9781, the linear regression equation is *F* = −57 876.7 + 109 999.3*C*_ATP_) in the range of 6–27 mM, and it is found that the limit of detection (LOD) is 5.0 μM, which was calculated based on 3*σ*_0_/*k* (*σ*_0_ is calculated using the standard deviation of the background, and *k* represents the slope of the calibration line). As shown in ESI Table S2,† the LOD of the proposed sensor is lower than that of previous sensors for ATP detection.^27,30,34,35^ The low LOD should be attributed to the ∼60% content of Ag NCs with a <2 nm average diameter. Thus, the aptasensor is shown to be highly sensitive. Moreover, the response time of the sensor is only 35 minutes, which is superior to that of the previously reported sensors.^30^

Furthermore, the fluorescence lifetimes of (L)BT5A5-Ag NCs in the absence and presence of various concentrations of ATP were evaluated with an emission wavelength of 620 nm (ESI Fig. S8†).

The fluorescence transients of (L)BT5A5-Ag NCs exhibit haploid exponential time constants (ESI Table S3†) with an average lifetime of 4.3 ns. The fluorescence lifetimes of (L)BT5A5-Ag NCs gradually decrease with increasing concentration of ATP. Therefore, the results illustrate that the interaction between ATP and (L)BT5A5-Ag NCs is a dynamic process.

### 3.5 Selectivity of the aptasensor toward ATP

**Fig. 5:**
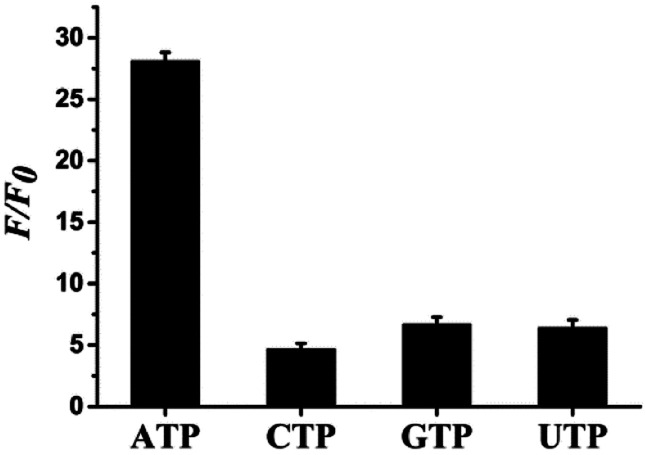
Specificity evaluation of the proposed sensor for ATP and its three analogues. The concentrations of ATP, GTP, CTP, and UTP are all 10 mM. Error bars represent the standard deviation of three parallel experiments.

Selectivity is an important parameter to evaluate the property of fluorescence sensors. Therefore, ATP and some other interfering species such as GTP, UTP, and CTP were tested under the same conditions to assess the specificity of the aptasensor for ATP detection. Fig. 5 shows that ATP causes a significant increase in *F*/*F*_0_, while the other analogues hardly affect it, thereby indicating a high specificity of the aptasensor strategy toward the target.

### 3.6 Detection of ATP in actual samples

The proposed aptasensor was employed to analyze ATP in actual samples. Solutions of 6, 9, 12, and 15 mM ATP were separately mixed with 100-times-diluted fetal bovine serum and measured using the proposed sensor following the same method. As presented in Table 1, the recoveries of this strategy are between 99.2% and 103.3%. In addition, the RSD values are in the range of 1.05–1.94%, which shows good precision. These results are comparable with previously reported values as well.^36,37^ Therefore, the detection results confirm the reliability of the aptasensor for detecting ATP in fetal bovine serum.

**Table 1:** The concentration of ATP in fetal bovine serum solution measured using the aptasensor (*N* = 3)

Samples	Spiked (mM)	Measured (mM) mean^a^ ± SD^b^	Recovery (%)	RSD^c^ (%)
1	6	6.2 ± 0.12	103.3	1.94
2	9	9.1 ± 0.13	101.1	1.43
3	12	11.9 ± 0.13	99.2	1.09
4	15	15.2 ± 0.16	101.3	1.05

a The mean of three determinations.

b SD = standard deviation.

c RSD = relative standard deviation.

## 4 Conclusions

In summary, a label-free fluorescence aptasensor has been constructed based on the fluorescence enhancement of DNA-Ag NCs, which can be used for the quantitative determination of ATP in the range from 6 to 27 mM with a LOD of 5.0 μM. Furthermore, it was successful in detecting ATP in fetal bovine serum. The proposed aptasensor possesses the advantages of sensitivity, specificity, simplicity, inexpensiveness, low toxicity, and avoiding chemical modification. Therefore, the proposed aptasensor can be applied in clinical diagnosis and the detection of biological samples, but further studies are still needed to develop a more sensitive sensor for ATP detection in real samples, and our group is working hard towards this objective.

## Conflicts of interest

There are no conflicts to declare.

## Supplementary Material

RA-012-D2RA04636A-s001
