# Supplementary material for: A label-free aptasensor for the detection of ATP based on turn-on fluorescence DNA-templated silver nanoclusters
Source: RSC Adv. 2022 Oct 20;12(46):30024–9. doi: 10.1039/d2ra04636a (PMC9582908; doi:10.1039/d2ra04636a)
Supplement: RA-012-D2RA04636A-s001 [file RA-012-D2RA04636A-s001.pdf]

Electronic Supplementary Material (ESI) for RSC Advances.
This journal is © The Royal Society of Chemistry 2022

Electronic Supplementary Information

A label-free aptasensor for the detection of ATP based on turn-on fluorescent DNA-template silver nanoclusters

Baozhu Zhang*, Ziyao Yang, Yuxia Li, Ma Ling, Fenfang Li, Xiuqing Lv, Guangming Wen*

Department of Chemistry and Chemical Engineering, Jinzhong University, Yuci 030619, P. R. China

*Corresponding author.

E-mail address: zhangbaozhu518@126.com; wgm@sxu.edu.cn

Table S1 Names and sequences of the oligonucleotides.

Oligonucleotids	Sequences (5´- 3´)
BT5A5	**CCCTTAATCCCC**TTTTT*AACCTGGGGGAGTATTGCGGAGGAAGGT*<br>AAAAA**CCCTAACTCCCC**
BT5A5(R)	**CCCTTAATCCCC**TTTTT*AACCTGG GGGAGTATTGCGGAGGAAGGT*
(L)BT5A5	*AACCTGGGGGAGTATTGCGGAGGAAGGT*AAAAA**CCCTAACTCCCC**

Table S2 Comparison of different strategies for the detection of ATP.

Detection methods	LOD	Linear range	References
DNA-Ag NC fluorescence light-up system	0.44 mM	0-4 mM	30
Fluorescence-based core–shell Ag@SiO_2 nanoflares	8 μM	0-500 μM	34
Light-up DNA-scaffolded silver nanoclusters	0.81 mM	1-6 mM	35
Fluorescence DNA-Cu/Ag NCs	7.0 μM	2-18 mM	27
Fluorescence DNA-Ag NCs	5.0 μM	6-27 mM	this work

Table S3 The lifetimes of (L)BT5A5-Ag NCs in the absence and presence of different concentration of ATP.

Samples	[ATP] (mM)	τ (ns)	χ^2
(L)BT5A5-Ag NCs	0	4.571	1.332
	3	4.317	1.313
(L)BT5A5-Ag NCs + ATP	6	4.195	1.336
	9	4.084	1.333

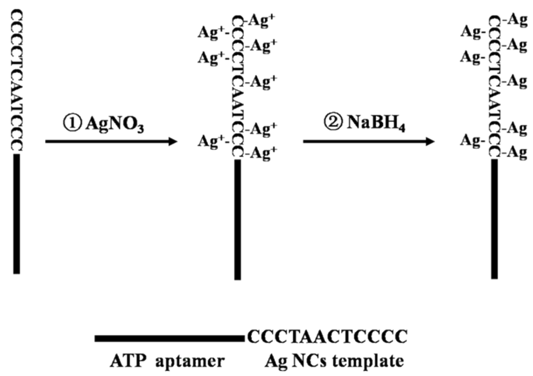

Fig. S1 Schematic illustration of synthesis of Ag NCs based DNA templates.

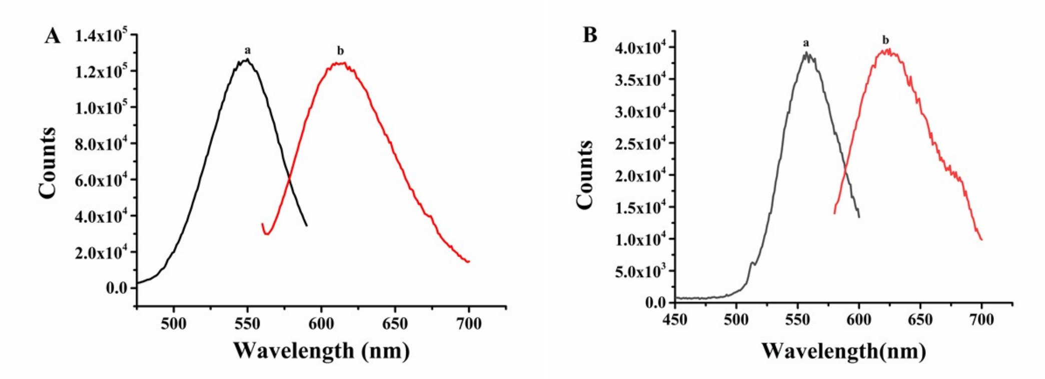

Fig. S2 (A) The excitation (a) and emission (b) spectra of BT5A5-Ag NCs. (B) The excitation (a) and emission (b) spectra of BT5A5(R)-Ag NCs.

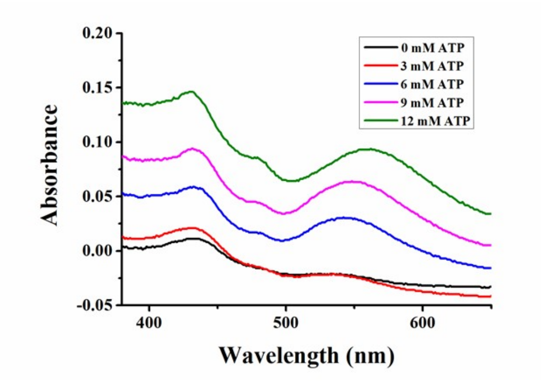

Fig. S3 UV-Vis absorption spectra of (L)BT5A5-Ag NCs under different concentrations of ATP.

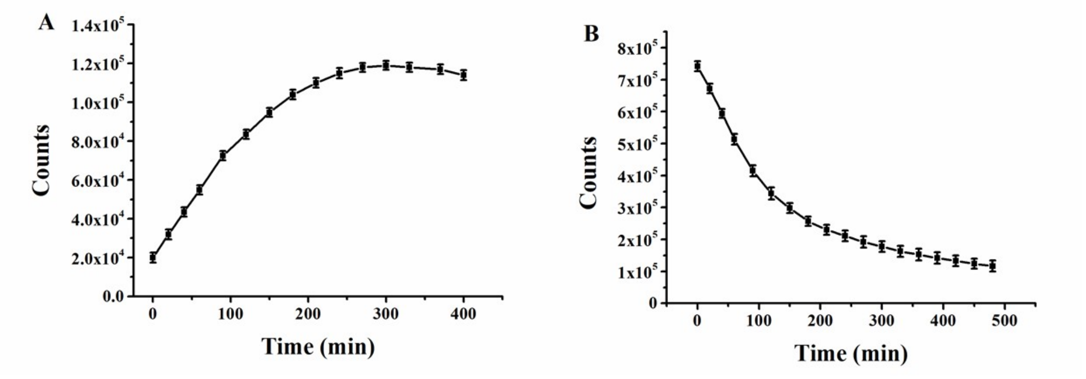

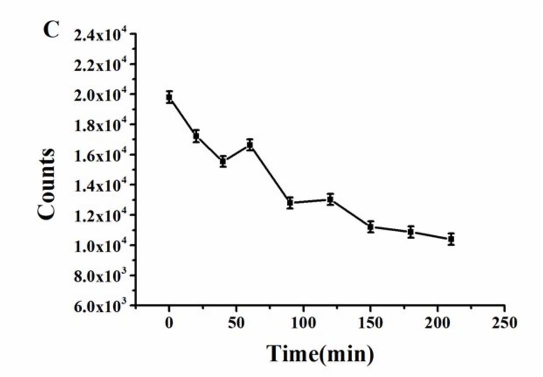

Fig. S4 The change of fluorescence intensity of (L)BT5A5-Ag NCs (A), BT5A5-Ag NCs (B), and BT5A5(R)-Ag NCs(C), *against* the increasing time. Error bars represent the standard deviation of three independent measurements. c(DNA) = 3.0 μM.

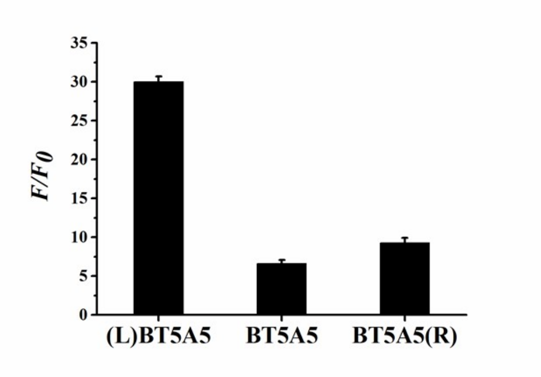

Fig. S5 Relative fluorescence intensity (F/F_0) of different DNA-Ag NCs. F_0 and F are the maximum emission intensity of the DNA-Ag NCs before and after the addition of 21 mM ATP, respectively. The error bars represent the standard deviation of three independent measurements.

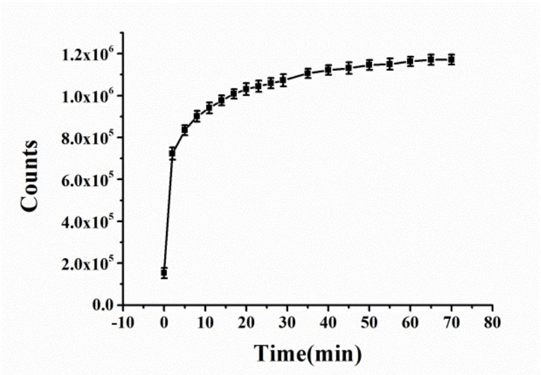

Fig. S6 Fluorescence intensity of (L)BT5A5-Ag NCs as a function of incubation time of (L)BT5A5-Ag NCs and ATP.

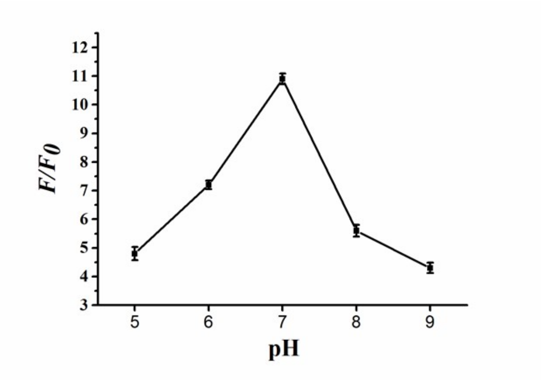

Fig. S7 Relative fluorescence intensity (F/F_0) of (L)BT5A5-Ag NCs at different pH values. F_0 and F are the maximum emission intensity of (L)BT5A5-Ag NCs without and with 12 mM ATP, respectively. The error bars represent the standard deviation of three independent measurements.

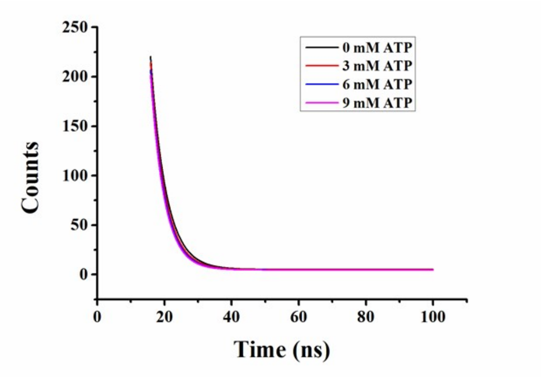

Fig. S8 The fluorescence lifetimes of (L)BT5A5-Ag NCs (excitation at 405 nm and emission at 620 nm) incubating without and with the different concentration of ATP.
